# Effect of root surface conditioning agents for enhancing periodontal regeneration

**DOI:** 10.6026/973206300210879

**Published:** 2025-04-30

**Authors:** Kaushal Pati Tripathi, Rohit Mishra, Varsha Choubey, Sukirti Singh, Yogesh Goswami, Aarohi Aglawe

**Affiliations:** 1 Department of Periodontics & Implantology, Hitkarini Dental College & Hospital, Jabalpur, Madhya Pradesh, India; 2 Department of Periodontics, Maitri College Of Dentistry And Research Center, Anjora, Durg, Chhattisgarh, India; 3 Department of Periodontology, Mansarovar Dental College, Kolar Bhopal, Madhya Pradesh, India

**Keywords:** Root surface, conditioning, periodontal regeneration

## Abstract

The surface characteristics of periodontal disease extracted human teeth after treatment with different root conditioning agents for periodontal regeneration is of interest. Hence, 80 specimens were randomly distributed among the treatment categories of ethylenediaminetetraacetic acid, mixture of Doxycycline, citric acid and a detergent, citric acid and tetracycline, with each category consisting of 20 specimens. Analysis show significant variations in dentin percentage (%), tubular space percentage (%) and tubular diameter (µm^2^) after treatment with all experimental root conditioning agents. The results were significantly better in the ethylenediaminetetraacetic acid and tetracycline groups, followed by the mixture of Doxycycline, citric acid and a detergent and citric acid groups. Ethylenediaminetetraacetic acid, mixture of Doxycycline, citric acid and a detergent, citric acid and tetracycline can be used successfully as root conditioning agents enhancing periodontal regeneration. However, ethylenediaminetetraacetic acid and tetracycline showed greater promising results.

## Background:

Evident renewal of the periodontal tissue in regions previously impacted by periodontal illness is one of the objectives of treatments for periodontal disease [1, 2- 3]. In addition to infection by various bacterial strains and accompanying endotoxins, the surfaces of roots may be susceptible to hyper-mineralization [4, 5- 6]. Because contamination or infection of the root surface may alter the results of regenerative periodontal treatments, infected root surfaces must be modified and disinfected to achieve the best possible outcome [7, 8-9]. Scaling and root planning (SRP), which eliminates calculus beneath the gum line and disinfects the root surface to facilitate the regeneration process, are the least intrusive methods for clearing the visible surface of roots from endotoxins, accretions and bacterial deposits [10, 11-12]. Nevertheless, the possible drawbacks of SRP have also been discussed. First, it appears that it is impossible to completely decontaminate the periodontitis-affected root surfaces with this treatment [13, 14- 15]. In actuality, scaling and root planing only address the periodontal disease temporarily. Furthermore, it has been demonstrated that these techniques are less successful in deeper pockets where it is more challenging to remove the calculus [16, 17-18]. The posterior teeth, which are difficult to access for manual root surface cleansing, are likewise affected. Root conditioning may be used as a supplement to mechanical debridement to address this issue [18, 19- 20]. Numerous materials and reagents have been developed to remove bacterial endotoxins, including the smear layer, from the surface of the root. Administration times have been evaluated between 0.5 and 10 minutes, with the majority of studies finding that 3 to 4 minutes produce the most significant outcomes [13, 14-15]. A published systematic evaluation of the topic concluded that there is no clinically significant improvement in regeneration in individuals with chronic periodontitis when the root surface is modified with EDTA, tetracycline and citric acid [16, 17- 18]. However, the author notes that a definitive conclusion should be approached with caution, as the majority of relevant research methods are not yet complete [19, 20- 21]. Searching for characteristics that can facilitate the adoption of this approach in periodontal treatments is justified by the ongoing and unresolved dispute surrounding the necessity of using chemical agents [15, 16-17]. Additionally, selecting the optimal application timing may be facilitated by understanding the effects of these medications over various time periods [12, 13, 14- 15]. Therefore, it is of interest to compare the surface characteristics of periodontal disease extracted human teeth after treatment with different root conditioning agents for periodontal regeneration.

## Materials and Methods:

For the current investigation, 80 single-rooted human teeth recommended for extraction due to chronic periodontitis were collected.

## Getting the specimen ready:

A soft-bristled brush, along with distilled water, was used to remove blood and saliva from the excised teeth. Following washing, a Gracey curette was used delicately for root planing of the root surfaces. Afterward, a high-frequency handpiece with a refining bur running at almost 400,000 revolutions per minute was applied to remove the cementum and provide an even glass-like, rigid surface. The tooth crowns were removed in each tooth at the level of the cementoenamel junction (CEJ) to create an experimental interface. A section of the root located 5 mm below the CEJ had been subsequently chosen. The specimen was cut into two identical longitudinal segments across the pulp chamber using a two-sided diamond disk set mounted on a handpiece operating at low speed that was continuously and copiously irrigated with water. The pulpal side was flattened with a straight bur and a vertical groove was created on the horizontal region for identification. The specimens were randomly distributed to one of the following treatment categories. Each category consisted of 20 specimens.

Category 1 - treated with Ethylenediaminetetraacetic Acid (EDTA) 24% (pH = 7.3)

Category 2 - treated with MTAD (MTA +Doxycycline)

Category 3 - treated with doxycycline 10% (pH = 2.2)

Category 4 - treated with tetracycline HCL 10% (pH = 1.8)

## Application of the root conditioning agents:

The experimental chemical reagents were applied to the root using a rubbing technique with cotton beads for 10 to 15 seconds and then left in place for 1 minute, 2 minutes, 3 minutes and 4 minutes. The tooth samples were subsequently rinsed with filtered water in order to halt the reactions. The samples were then allowed to air dry after being dehydrated in alcohol solutions with increasing concentrations of alcohol.

## Specimen preparation for SEM study:

Following treatment, the root surfaces were preserved for twenty-four hours at 40 degrees Celsius in a phosphate buffer with a pH of 7.3 and 2.5 percent glutaraldehyde. The specimens were set aside in osmium tetroxide in 1.5% phosphate buffer for two hours and then they were rinsed three times in phosphate buffer for ten minutes each. After that, the samples were dried for 10 minutes each in a graded series of ethanol solutions. The materials were dehydrated overnight in a silicone gel desiccator jar, followed by two further 10-minute rinses in 100% ethanol. The specimens were mounted on silver-painted SEM stubs. A scanning electron microscope (SEM) was used to view and analyze each sample. After being transferred to a computer, the SEM micrographs were examined using Image J software. The field displayed at 1,000x magnification was used as a reference for the entire region. The mean diameter of the patent dentinal tubules, based on a 10 µm scale bar, as well as the percentage of areas filled by dentin and the expanded dentinal tubules, were computed.

## Statistical analysis:

The one-way ANOVA test was used to assess intergroup differences and changes in study indices following interventions.

## Results:

It was observed that there was an increase in tubular space (%) after treatment with all root conditioning agents. However, the increase was maximum in EDTA group (43.8±0.7% to 53.1±1.3%) and tetracycline group (42.1±0.9% to 54.4±0.5%) followed by MTAD (43.1±0.6% to 49.4±0.7%) and citric acid (43.0±0.7% to 45.2±0.4%). The findings were significant statistically (Table 1). There were significant variations in dentin (%) after treatment in all experimental root conditioning agents. The variation was maximum in ethylenediaminetetraacetic acid (EDTA) group (58.4±0.7% to 51.1±1.3%) and tetracycline group (60.1±0.9% to 47.8±0.5%) followed by MTAD (59.1±0.6% to 52.7±0.7%) and citric acid (58.2±0.7% to 56.9±0.4%). The findings were significant statistically (Table 2). It was observed that there was an increase in tubular diameter (µm^2^) after treatment with all root conditioning agents. However, the increase was maximum in the EDTA group (4.6±0.6 µm^2^ to 7.7 µm^2^) and tetracycline group (4.4±0.6µm^2^ to 7.9±0.9µm^2^), followed by MTAD (mixture of Doxycycline, citric acid and a detergent) (4.4±0.6 µm^2^ to 8.2±0.6 µm^2^) and citric acid (5.7±0.6µm^2^ to 4.4±0.7µm^2^). The findings were significant statistically (Table 3).

## Discussion:

To eliminate bacterial endotoxins, including the smear layer from the root surface, various materials and reagents have been developed. The author does, however, note that since most of the pertinent study methodologies are yet incomplete, a conclusive conclusion should be taken into consideration [22-23]. The on-going debate about the necessity of using chemical agents justifies the search for traits that can facilitate the adoption of this strategy in periodontal treatments [24, 25- 26]. Additionally, knowing how these medications work across different time periods may help determine the best time to apply them [22, 23- 24]. Therefore, the present study aimed to compare the surface characteristics of periodontal disease extracted human teeth after treatment with different root conditioning agents. In our study, there was an increase in tubular space (%) after treatment with all root conditioning agents. However, the increase was maximum in ethylenediaminetetraacetic acid group (43.8±0.7% to 53.1±1.3%) and tetracycline group (42.1±0.9% to 54.4±0.5%) followed by MTAD (43.1±0.6% to 49.4±0.7%) and citric acid (43.0±0.7% to 45.2±0.4%). The findings were significant statistically. The findings of our study are consistent with those of other studies, which have also reported significant improvements in tubular space indicators using EDTA and Tetracycline as root conditioning agents [22, 23- 24]. Some studies also reported positive results with MTAD, as well as citric acid, as root conditioning agents [25, 26-27]. One goal of periodontal disease therapies is to clearly restore periodontal tissue in areas that were previously affected by the disease [11, 12-13]. The surfaces of roots may be vulnerable to hyper-mineralization in addition to infection by different bacterial strains and associated endotoxins [14, 15-16]. For optimal results, the infected root surfaces must be modified and cleansed, as contamination or infection of the root surface can alter the outcome of regenerative periodontal treatments [17, 18- 19]. The least invasive technique for removing endotoxins, accretions and bacterial deposits from the visible root surface is scaling and root planning (SRP), which eliminates calculus beneath the gum line and cleans the root surface to promote regeneration [20, 21-22. In our study, significant variations in dentin percentage were observed after treatment with all experimental root conditioning agents. The variation was maximum in EDTA group (58.4 ± 0.7% to 51.1 ± 1.3%) and tetracycline group (60.1 ± 0.9% to 47.8 ± 0.5%) followed by MTAD (59.1 ± 0.6% to 52.7 ± 0.7% ) and citric acid (58.2 ± 0.7% to 56.9 ± 0.4%). This is statistically significant.

The findings of our study are consistent with those of other studies, which have also demonstrated significant improvements in indicators of remaining dentin using EDTA and Tetracycline as root conditioning agents [21, 22, 23-24]. Some studies also reported positive results with MTAD, as well as citric acid, as root conditioning agents [24, 25, 26-27]. However, there has also been a discussion of SRP's potential disadvantages. First, this treatment appears to be unable to fully clean the root surfaces damaged by periodontitis [23, 24]. In reality, scaling and root planing only provide short-term relief from periodontal disease. Additionally, it has been demonstrated that these methods are less effective in deeper pockets, where the calculus is more challenging to extract [25, 26-27]. This also affects the posterior teeth, which are difficult to reach for manual cleaning of the root surface. To overcome this issue, mechanical debridement may be supplemented with root conditioning [27]. In our study, there was an increase in tubular diameter (µm^2^) after treatment with all root conditioning agents. However, the increase was maximum in EDTA group (4.6±0.6µm^2^ to 7.7µm^2^) and tetracycline group (4.4 ± 0.6µm^2^ to 7.9 ± 0.9µm^2^) followed by MTAD (4.4 ± 0.6 µm^2^ to 8.2 ± 0.6 µm^2^) and citric acid (5.7 ± 0.6µm^2^ to 4.4 ± 0.7 µm^2^). The findings were significant statistically. The observations of our study have been supported by other studies [19, 20, 21-22]. These studies also found significant improvement in indicators of tubular diameter with EDTA and Tetracycline as root conditioning agents [25, 26-27]. Some studies do not support our findings. According to a published systematic examination of the subject, modifying the root surface with EDTA, tetracycline and citric acid does not result in a clinically meaningful improvement in regeneration in people with chronic periodontitis [19, 20-21]. The author does, however, note that since most of the pertinent study methodologies are yet incomplete, a conclusive conclusion should be taken into consideration [20, 21-22]. Based on our results and a comparison with other research, the use of EDTA-even by itself-produced the best root conditioning outcomes. In actuality, the use of EDTA in clinical practice is quite advantageous due to its natural pH and ability to eliminate the smear layer at the root surface [16, 17, 18-19]. Our findings suggest that tetracycline may be used as a backup option for root conditioning. Numerous studies have assessed and compared tetracycline's effectiveness with that of other substances, such as citric acid [21, 22]. The results of a survey showed that citric acid and EDTA test samples had a significantly greater number of patent tubules than the tetracycline hydrochloride testing category [25, 26-27].

## Conclusion:

Ethylene-di-amine-tetra-acetic acid, mixture of doxycycline, citric acid and a detergent, citric acid and tetracycline can be used successfully as root conditioning agents enhancing periodontal regeneration. However, ethylene-di-amine-tetra-acetic acid and tetracycline showed greater promising results.

## Figures and Tables

**Table 1:** Variations in tubular space in different root conditioning agents

	**Category 1 EDTA**	**Category 2 MTAD**	**Category 3 Citric acid**	**Category 4 Tetracycline HCL**	**t value**	**P value**
**Tubular space (%)**						
Preoperative values	43.8±0.7	43.1±0.6	43.0±0.7	42.1±0.9	21.231	0.021
Postoperative values	53.1±1.3	49.4±0.7	45.2±0.4	54.4±0.5		
t value		20.224				
P value		0.0321				

**Table 2:** Variations in dentin after treatment with different root conditioning agents

	**Category 1 EDTA**	**Category 2 MTAD**	**Category 3 Citric acid**	**Category 4 Tetracycline HCL**	**t value**	**P value**
**Dentin (%)**						
Preoperative values	58.4±0.7	59.1±0.6	58.2±0.7	60.1±0.9	22.342	0.011
Postoperative values	51.1±1.3	52.7±0.7	56.9±0.4	47.8±0.5		
t value		19.863				
P value		0.0421				

**Table 3:** Variations in tubular diameter after treatment with different root conditioning agents

	**Category 1 EDTA**	**Category 2 MTAD**	**Category 3 Citric acid**	**Category 4 Tetracycline HCL**	**t value**	**P value**
**Tubular diameter (µm^2^)**						
Preoperative values	4.6±0.6	4.4±0.6	5.7±0.6	4.4±0.6	22.32	0.02
Postoperative values	7.7±0.5	8.2±0.6	4.4±0.7	7.9±0.9		
t value		21.113				
P value		0.021				
